# The Safety and Efficacy of Low-Molecular-Weight Heparin in Pregnant Women With Rheumatic Heart Disease and Valves Replacement

**DOI:** 10.7759/cureus.23052

**Published:** 2022-03-11

**Authors:** Najwa Alghamdi, Saeed Alqahtani, Lujain Allehyani, Haifa Alosaimi, Waleed Almutairi, Saleh Alobaid, Hanan B Albackr, Latifah Aldakhil, Ghazi S Alotaibi, Farjah H Alqahtani

**Affiliations:** 1 Department of Clinical Pharmacy, Adham General Hospital, Jeddah, SAU; 2 Department of Clinical Pharmacy, College of Pharmacy, King Saud University, Riyadh, SAU; 3 Department of Medicine, Alfaisal University College of Medicine, Riyadh, SAU; 4 Department of Cardiovascular Medicine, King Saud University, Riyadh, SAU; 5 Department of Obstetrics and Gynaecology, College of Medicine, King Saud University, Riyadh, SAU; 6 Department of Hematology, College of Medicine, King Saud University Medical City, Riyadh, SAU; 7 Department of Medicine, King Saud University, Riyadh, SAU

**Keywords:** heart valve replacement, heparin, lmwh, pregnancy, rheumatic heart disease, warfarin

## Abstract

Background: In patients with rheumatic heart disease (RHD) and prosthetic valve replacement, the risk of thromboembolic complications is the highest during and immediately after pregnancy. Therapeutic anticoagulation during this period is crucial to minimize the risk of thromboembolic complications. The use of low-molecular-weight heparin (LMWH) remains an off-label indication. The type of anticoagulants used, dosing regimens, target anti-Xa levels, and frequency of anti-Xa monitoring are highly variable in the pregnant population and have been derived from pilots, observational studies, and empirical evidence. Herein, in a real-world setting, we sought to examine the efficacy and safety of variable anticoagulation options with a focus on LMWH in the management of RHD-related valvular disease in pregnant women.

Methods: This study is a retrospective study conducted at a large university-affiliated tertiary care center (King Saud University Medical City) between January 2011 and February 2020. All pregnant women with RHD who had heart valve replacements were reviewed. Patient data were extracted for demographic information, baseline characteristics, anticoagulation type, and primary outcomes. Primary endpoints were thromboembolic events, hemorrhagic complications, and fetal outcomes.

Results: A total of 744 pregnancies in 149 women were identified. The mean age ± SD of the women was 43.8 ± 12 years. A total of 86 women (58%) were on the LMWH regimen, 35 women (23%) were on LMWH and warfarin regimen, and 28 women (19%) were on unfractionated heparin (UFH) and warfarin regimen. Overall, thromboembolic events developed in five (0.7%) pregnancies. Of those, two were in the LMWH group, two were in the LMWH and warfarin group, and one was in the UFH and warfarin group. In addition, significant hemorrhagic complications occurred in five pregnancies. Of these, two occurred in the LMWH group, two in the LMWH and warfarin group, and one in the UFH and warfarin group. No adverse maternal and fetal outcomes were noted.

Conclusion: This study presents the largest retrospective study of variable anticoagulation options in pregnant women with RHD and prosthetic valve replacement. LMWH is both safe and effective in preventing major thromboembolic complications compared to other forms of anticoagulation used during pregnancy.

## Introduction

Rheumatic heart disease (RHD) is a consequence of improper host immune response to a beta-hemolytic streptococcal infection of the pharynx [1]. Rheumatic valvular disease predominates in resource-limited countries, accounting for 50-90% of all cardiovascular morbidities in pregnancy, necessitating valve replacement with either mechanical or bioprosthetic valves, or valve repair [2,3]. The use of anticoagulation is indicated following surgery to reduce the risk of cardioembolic events in patients [4]. Anticoagulation choices in this setting include vitamin K antagonists (VKA) such as warfarin, unfractionated heparin (UFH), and low-molecular-weight heparin (LMWH) [4]. Direct oral anticoagulants are associated with increased rates of thromboembolic and bleeding complications, as compared with warfarin [5].

Pregnancy is a hypercoagulable state and the risk of thromboembolic complications is higher in pregnancy [6,7]. Maintenance of therapeutic anticoagulation is needed to minimize the risk of thromboembolic complications in pregnant patients with RHD [8]. The benefits and risks of various anticoagulation regimens should be extensively discussed with the patients before pregnancy [9].

In non-pregnant women with mechanical heart valves, warfarin is considered the most effective option for the prevention of thromboembolic complications; however, due to its placental penetration, it can result in teratogenic effects when used during pregnancy [8,10]. Substitution of warfarin with UFH or LMWH at least during the first trimester is indicated [11].

In this study, we investigated the efficacy and safety of LMWH in the management of RHD-related valvular disease in pregnant women.

## Materials and methods

Study design and subjects

This is a retrospective cohort study conducted at one of the largest academic tertiary care facilities in Saudi Arabia (King Saud University Medical City) between January 1, 2011, and February 28, 2020. We included all pregnant women with RHD who had a heart valve replacement. Information retrieved from medical records included demographic data, indication for and date of valve replacement, type of valve replacement, valve site, anticoagulation used, evidence of cardiac failure on cardiac imaging, and the presence of risk factors for thromboembolic events. These included thrombogenic mechanical valves such as two mechanical valve replacements, history of previous thromboembolism (valve thrombus, transient ischemic attack (TIA), and cerebrovascular accident (CVA)), atrial ﬁbrillation, and venous thrombosis. Data were collected on anticoagulation therapy (warfarin, enoxaparin, and UFH), including the dose, gestational period during which treatment was prescribed, peak anti-Xa levels (whilst on LMWH), international normalized ratio (INR) (whilst on warfarin), and detailed information about peripartum anticoagulation bridging. The primary endpoints, thromboembolism, and hemorrhagic complications were recorded. Diagnosis of valve thrombus required conﬁrmation on echocardiogram. Fetal/neonatal outcome measures included live birth rate, abortion, miscarriage, stillbirth, and neonatal death.

Anticoagulation treatment

Reviewed patients were on one of the following regimens: (1) LMWH throughout pregnancy; (2) LMWH and transitioned to warfarin in the second trimester until pre-delivery; and (3) unfractionated heparin transitioned to warfarin from the second trimester onward. In the last two regimens, LMWH or UFH were given from 10 to 12 weeks of gestation, followed by warfarin substitution for the majority of the pregnancy until 36 weeks of gestation, when LMWH or heparin was then resumed. LMWH was initially administered as a twice-daily dose of 1 mg/kg. The dose is adjusted based on peak anti-Xa levels. Pregnant women in the warfarin group continued to take warfarin from the second trimester until 36 weeks of gestation when warfarin was substituted with LMWH or heparin to reduce the risk of hemorrhagic complications. The dose of warfarin is adjusted to maintain a valve-specific target INR of 2.5-3.5.

Statistical analysis

Descriptive analysis (e.g. mean ± standard deviation or ‎median quartile for continuous variables and frequency or ‎percentage for categorical ‎variables) was conducted for baseline characteristics. Chi-square test (two-sided) ‎and Fisher’s exact test (two-sided) were used to compare categorical variables and ‎Student’s t-test for continuous variables. Incidence rates were calculated as ‎cumulative incidence and compared using the ‎hazard ratios and corresponding 95% confidence intervals. SPSS software (version 26; IBM Corp., Armonk, NY) was used in this analysis.

## Results

Subject characteristics

A total of 744 pregnancies in 149 women were identified. Maternal characteristics, risk factors for thromboembolic events, and anticoagulant regimens during the pregnancies are shown in Table 1. The mean age ± SD of the women was 43.8 ± 12 years. A total of 86 women (58%) were on the LMWH regimen, 35 women (23%) were on LMWH and warfarin regimen, and 28 women (19%) were on UFH and warfarin regimen. A total of 101 women (68%) had a prosthetic valve replacement, while 48 women (32%) had a tissue valve replacement. Of these pregnancies, 19% (n = 139) occurred in women with aortic valves, 61% (n = 455) occurred in women with mitral valves, 2% (n = 15) occurred in women with tricuspid valves, and 18% (n = 135) had both aortic and mitral mechanical valves.

**Table 1:** Maternal characteristics and thromboembolic risk factors. LMWH, low-molecular-weight heparin.

Characteristics	
Maternal characteristics, mean (SD)	
Age, years	43.8 (12)
Weight, kg	68.8 (16.3)
Height, cm	156 (6.3)
Types of valves, % (n)	
Aortic valves	19% (n = 139)
Mitral valves	61% (n = 455)
Tricuspid valves	2% (n = 15)
Aortic and mitral mechanical valves	18% (n = 135)
Anticoagulation during pregnancy, n (%)	
LMWH regimen	86 (58%)
LMWH and warfarin	35 (23%)
Heparin and warfarin	28 (19%)
Thromboembolic risk factors, n (%)	
Previous thromboembolism	10 (6.7)
Two mechanical valves	27 (18%)
Atrial fibrillation	11 (7.5%)
≥ 1 thromboembolic risk factor	31 (21%)

Of patients in the LMWH group, 62% had prosthetic valves and the remaining had tissue valves (Table 2). The percentage of patients who had prosthetic valves was higher (71%) in the LMWH and warfarin group. Furthermore, 79% of the patients in the UFH and warfarin group had prosthetic valves. The number of patients who had prosthetic valves was significantly higher than tissue valves in all three groups (p < 0.01).

**Table 2:** Type of valve (tissue or prosthetic) in each treatment group. LMWH, low-molecular-weight heparin.

Treatment group		Type of valve		
	Overall (N = 149)	Tissue	Prosthetic	P-value
LMWH regimen	86 (58%)	32 (37%)	54 (63%)	0.002
LMWH and warfarin	35 (23%)	10 (29%)	25 (71%)	0.001
Heparin and warfarin	28 (19%)	6 (21%)	22 (79%)	0.001

Maternal outcome

Thrombosis Complications

Overall, thromboembolic compilations developed in five (0.7%) pregnancies; of these, two were in the LMWH group, two were in the LMWH/warfarin group, and one in the heparin/warfarin group (Table 3). All these thromboembolic complications occurred in the patients who had prosthetic tissue valves. For the two pregnancies in the LMWH group, the anti-Xa levels were within the therapeutic ranges and there were no thromboembolic risk factors in these pregnancies. Whereas, for the two pregnancies in the LMWH and warfarin group, the anti-Xa levels were within the therapeutic ranges during the first trimester and treatment with LMWH. However, the INR levels were in the sub-therapeutic range for these pregnancies. The last case was treated with heparin and warfarin and had an INR level within the target range. Both patients on heparin and warfarin had mechanical valve replacements (mitral and aortic).

**Table 3:** Maternal thromboembolic and hemorrhagic complications and fetal outcomes (N = 744 pregnancies; 218 had tissue valves and 526 had prosthetic valves). * No events were documented in patients with tissue valves. LMWH, low-molecular-weight heparin; UFH, unfractionated heparin.

Maternal complications	All patients*		Prosthetic valve	
	Total events, number (%)	95% CI	N (%)	95% CI
Thromboembolic complications, total	5 (0.7)	0.2-1.8	5 (0.95)	(0.2-1.93)
LMWH regimen	2 (0.46)	0.1-0.95	2 (0.66)	(0.1-0.92)
LMWH and warfarin	2 (1.22)	0.5-1.65	2 (1.74)	(0.4-1.86)
UFH and warfarin	1 (0.7)	0.1-1.1	1 (0.9)	(0.3-1.1)
Hemorrhagic complications, total	5 (0.7)	0.2-1.8	5 (0.95)	(0.2-1.93)
LMWH regimen	2 (0.46)	0.1-0.95	2 (0.66)	(0.1-0.92)
LMWH and warfarin	2 (1.22)	0.5-1.65	2 (1.74)	(0.4-1.86)
UFH and warfarin	1 (0.7)	0.1-1.1	1 (0.9)	(0.3-1.1)

Hemorrhagic Complications

Major bleeding occurred in five pregnancies. Of these, two occurred in the LMWH group, two in the LMWH and warfarin group, and one in the UFH and warfarin group (Table 3). Similar to the thromboembolic compilations, all of the hemorrhage events occurred in the patient who had prosthetic valves. Two of the three cases in warfarin groups had INR levels higher than the therapeutic target range. While the two cases in the LMWH had normal anti-Xa levels. Two women in the LMWH group were on aspirin. One of them had post-partum hemorrhage (PPH) and the other had a hematoma.

Fetal outcomes

The percentage of abortion was similar in LMWH (12.5%) and LMWH and warfarin (11.5%) groups. In contrast, the percentage of abortions in the UFH and warfarin group was 6% (Table 4). A similar observation was seen with miscarriage and stillbirth percentages across all groups. The rate of live birth was high in all groups (range: 85-93%). Although, it was slightly higher in the heparin and warfarin group.

**Table 4:** Fetal outcomes in all pregnancies per each treatment group. LMWH, low-molecular-weight heparin; UFH, unfractionated heparin.

Fetal outcomes	LMWH regimen (n = 432 pregnancies)	LMWH and warfarin (n = 164 pregnancies)	UFH and warfarin (n = 148 pregnancies)
Abortion	53 (12.5%)	19 (11.5%)	9 (6%)
Miscarriage	4 (0.9 %)	1 (0.6 %)	0 (0%)
Stillbirth	6 (1.4 %)	3 (1.83 %)	1 (0.7)
Live birth	366 (85%)	141 (86%)	138 (93%)

## Discussion

Physiological changes of pregnancy are associated with an increase in the risk of maternal and fetal complication events [6,7]. Patients of childbearing age with mechanical prosthetic valves pose unique challenges since there is no optimal anticoagulation agent considered completely safe at all stages of pregnancy [12]. Effective anticoagulation therapy is necessary for pregnant women who had received mechanical heart valve replacement [9]. Because of warfarin's teratogenic effects and its relation with a high rate of abortion, there is general agreement that warfarin is contraindicated during the first trimester of pregnancy [13].

Here, we reported a large number of pregnant women with RHD who had a heart valve replacement. Five thromboembolic events were reported in our cohort (0.7%, 95% CI: 0.2-1.8). Additionally, valve thrombosis occurred only in one patient in the LMWH and warfarin group. In this patient, the INR levels were sub-therapeutic. No valve thrombosis was reported in the LMWH/VKA or UFH/VKA groups. Vural et al. [14] reported a similarly low incidence of thromboembolic events in women who were compliant with anticoagulation. In contrast, several studies have reported a higher incidence of thromboembolic complications in pregnancy [13,15-17]. Rowan et al. and Yinon et al. reported the rate of valve thrombosis during LMWH treatment was 7%, while, Ayad et al. reported a higher rate of valve thrombosis with 7.5% [15-17]. Additionally, Geelani et al. found the incidence of valve thrombosis with heparin plus warfarin regimen was 1%, as a result of poor compliance with heparin therapy [13]. The occurrence of valve thrombosis in Yinon et al.'s study despite close monitoring of anti-Xa levels and therapeutic anti-Xa levels ranged from 1 to 1.4 U/ml [17]. Apart from this, patients' poor compliance was the reason in Rowan et al.'s study [16].

The reported rate of thromboembolic complications was lowest in the LMWH regimen compared to the other regimens. Despite two patients who had thromboembolic complications, their anti-Xa levels were within the therapeutic ranges and there were no thromboembolic risk factors in these pregnancies. On further review, the INR levels were in the sub-therapeutic range for these pregnancies in LMWH/warfarin regimen. Despite the INR level within the target range of one case who was on UFH/warfarin treatment, she developed a thrombosis. Importantly, she had a thromboembolic risk factor, which was a double valve replacement.

Compared to our result, the rates of maternal thromboembolic complications in the LMWH group were relatively low in McLintock et al.'s study, which was 10.6% [8]. This could be due to poor compliance, sub-therapeutic anti-Xa levels, or other factors [8].

Total hemorrhagic complications were reported in five pregnancies in our study (0.7%, 95% CI: 0.2-1.8). The lowest percentage of hemorrhage is documented in the LMWH regimen. Two pregnancies were complicated by PPH (0.46%, 95% CI: 0.1-0.95). In the UFH/warfarin regimen, one woman had an abdominal hematoma and epistaxis (0.7%, 95% CI: 0.1-1.1). In the LMWH/VKA arm, two pregnancies were complicated by fetal intracranial hemorrhage (ICH) and uterine bleeding. However, women in the LMWH group had a normal range of anti-Xa but all were on low-dose aspirin, which might have contributed to the high rate of bleeding.

Vause et al. noted the highest rate of bleeding in LMWH treatment with PPH complication (29%) [18]. In Yinon et al.'s study, 13% of patients had hemorrhagic complications, although anti-Xa levels were in the therapeutic range [17]. Moreover, McLintock et al. reported 12.7% PPH and stated that it could be related to low-dose aspirin [8]. In addition, higher rates than our result of the heparin plus warfarin were noted by Geelani et al. and Vural et al., with the rate of 2% and 5.3%, respectively [13,14]. While in studies by Rowan et al. and Vural et al., there was no bleeding tendency related to the LMWH regimen [14,16]. Also, the LMWH plus warfarin regimen in Rowan et al.'s study showed no bleeding complications [16].

UFH/warfarin regimen showed the lowest percentage of abortion and the highest rate of live birth while the opposite in the LMWH regimen as presented in Table 2. The percentage of live birth in LMWH in our cohort is 85%, which was greater than what has been reported in Ayad et al.'s study with 58.5% [15]. On the other hand, D’Souza et al. reported a higher percentage of live birth than our study with 92% [19]. We reported a stillbirth rate of 1.4% in the LMWH group, which was lower than what has been reported by McLintock et al. (5.7%) [8].

This study has some limitations. We retrospectively collected data on patients who were treated and followed in a large tertiary care hospital; however, outcomes that happened elsewhere are not captured, but we believe this should be equal for all groups. In addition, some of the outcomes could be attributed to poor compliance to the anticoagulation regimen used and, in this case, will not reflect the true safety of this therapeutic option. We believe the last factor is difficult to adjust for, but we reported the INR status when we reported adverse outcomes.

## Conclusions

This study presents the largest retrospective study of LMWH use in pregnant women with RHD who had a valve replacement. LMWH use among pregnant women is associated with successful pregnancy outcomes and the incidence of adverse thrombotic or hemorrhagic complications is low if patients are followed by high-risk obstetrics and monitored frequently. Similar fetal outcomes were noted in all three arms of the study. Further studies including randomized controlled trials investigating the use of LMWH for these indications are encouraged.
