# Correction: The Safety and Efficacy of Low-Molecular-Weight Heparin in Pregnant Women With Rheumatic Heart Disease and Valves Replacement

**DOI:** 10.7759/cureus.c366

**Published:** 2025-10-17

**Authors:** Najwa Alghamdi, Saeed Alqahtani, Lujain Allehyani, Haifa Alosaimi, Waleed Almutairi, Saleh Alobaid, Hanan B Albackr, Latifah Aldakhil, Ghazi S Alotaibi, Farjah H Alqahtani

**Affiliations:** 1 Department of Clinical Pharmacy, Adham General Hospital, Jeddah, SAU; 2 Department of Clinical Pharmacy, College of Pharmacy, King Saud University, Riyadh, SAU; 3 Department of Medicine, Alfaisal University College of Medicine, Riyadh, SAU; 4 Department of Cardiovascular Medicine, King Saud University, Riyadh, SAU; 5 Department of Obstetrics and Gynaecology, College of Medicine, King Saud University, Riyadh, SAU; 6 Department of Hematology, College of Medicine, King Saud University Medical City, Riyadh, SAU; 7 Department of Medicine, King Saud University, Riyadh, SAU

This article has been revised to update the affiliation for Lateefa Aldakhil. The previous affiliation was: ​​ 

- Department of Obstetrics and Gynaecology, King Saud University Medical City, Riyadh, SAU

This has been clarified as:

- Department of Obstetrics and Gynecology, College of Medicine, King Saud University, Riyadh, Saudi Arabia

